# Differential Modulation of Human Innate Lymphoid Cell (ILC) Subsets by IL-10 and TGF-β

**DOI:** 10.1038/s41598-019-50308-8

**Published:** 2019-10-04

**Authors:** Sandra Bonne-Année, Mabel C. Bush, Thomas B. Nutman

**Affiliations:** Helminth Immunology Section, Laboratory of Parasitic Diseases, National Institute of Allergy and Infectious Diseases, National Institutes of Health, 9000 Rockville Pike, Bethesda, MD 20892 USA

**Keywords:** Cytokines, Innate lymphoid cells

## Abstract

Using multiparameter flow cytometry human innate lymphoid cell (ILC) subsets can be detected in the circulation, in relatively low frequencies. Despite the low frequency of ILCs in circulation, *ex vivo* experiments have demonstrated that these ILCs release extremely large per cell quantities of signature ILC cytokines following activation. To determine how activated ILC cytokine production is regulated, ILC subsets were activated in the presence or absence of the immunoregulatory cytokines IL-10 and TGF-β. An examination of circulating ILC subsets revealed surface expression of IL-10Rα and mRNA expression of both IL-10Rα and TGF-βR1 for all ILC subsets. Stimulated ILC1 production of IFN-γ was decreased by TGF-β and not IL-10. Interestingly, ILC2s stimulated in the presence of IL-10 had a marked reduction in cytokine production of IL-5 and IL-13 while TGF-β had no effect on ILC2 cytokine production. *Ex vivo* activated ILC1 and ILC2 subsets were also found to be a source of the immunoregulatory cytokine IL-10, raising the potential for ILC-mediated regulation of immune cells. These findings demonstrate the differential effects of immunoregulatory cytokines IL-10 and TGF-β on activated ILC1 and ILC2 populations *ex vivo*.

## Introduction

Innate lymphoid cells (ILCs) can be found in both tissues and in peripheral blood in healthy individuals, but changes in their frequencies and composition have been associated with a variety of human diseases^1^. Human ILC populations include cytotoxic ILCs (NK cells), helper ILCs and the recently identified regulatory ILCs (ILCreg). Helper ILCs consist of 3 phenotypically distinct subsets: ILC1s (Lin-CD45+ CD127+ cKit-NKp44−), ILC2s (Lin-CD45+ CD127+ CRTH2+) and ILC3s (Lin-CD45+ CD127+ cKit + NKp44−/+) with each subset responding to cytokine activation by producing IFN-γ (for ILC1s), IL-5 and IL-13 (for ILC2s) and IL-17A or IL-22 (for ILC3s). Human ILCreg are phenotypically described as Lin-CD45+ CD127+ IL-10+ cells found in intestinal tissue and express a distinct cytokine gene profile that includes IL-10 and TGF-β1^2^.

Although found in variable frequencies, helper ILC subsets have clearly been implicated in driving certain immunological responses owing to their production of specific cytokines namely IFN-γ, TNF-α, IL-13 and IL-17A. ILC2s have been implicated as mediators of parasite clearance^3,4^ through the production of IL-5 and IL-13 and in the context of allergic disease expansion of ILC2s help to explain tissue eosinophilia^5^, increased mucus production^6^ and airway hyper-reactivity^7^. IL-22-producing ILC3s exert antimicrobial effects while also promoting tissue repair and remodeling in the gut^8,9^. These same ILC3s serve as a major contributor to the pathogenesis of psoriasis and rheumatoid arthritis^10^. ILC1s are greatly expanded in the gut tissue of individuals with Crohn’s disease^11^ and have been shown to contribute to obesity-related pathology such as insulin resistance in mice through the production of IFN-γ^12^. However, the contributions of ILC1s to disease pathology have not been well-defined, given the predominance of other IFN-γ producing cells^13–15^.

Modulation of the immune response is a multifactorial process that is mediated by signaling through cellular receptors for the immune checkpoint regulators^16^ such as program cell death-ligand 1 (PD-L1)^17,18^, cytotoxic T cell lymphocyte antigen-4 (CTLA-4), and inducible T cell costimulator (ICOS)) as well as the receptors for IL-10 and TGF-β. The release of IL-10 and TGF-β is a trademark of regulatory T-cells^19^, B-cells^20^, myeloid- cells^21^ and innate lymphoid cells^2^. Although regulatory networks are critical to the control of inflammation induced by innate and adaptive cells, little is known about immune regulation of ILCs. One known regulator, PD-1, which, expressed on all murine ILC subsets^22^, is critical for the modulation of KLRG1+ ILC2s in mice, but also for the effector function of human ILC2s (the only human ILC subset shown to express PD-1^23^). Inducible Tregs have been shown to modulate the effector function of human ILC2s in a humanized mouse model through both ICOS/ICOS ligand interactions and through the effects of IL-10 and TGF-β^24^. Nevertheless, how cytokine production is regulated in human ILCs has not been fully elucidated.

The present study attempts first to understand the composition of ILCs in the circulation, to characterize the cytokine profile of activated human ILC subsets *ex vivo* and to understand the mechanisms used to modulate ILC effector function. Whole blood flow cytometry was first utilized to assess the frequency and compositions of ILC subsets at homeostasis with minimal perturbation. The same flow cytometric approach was utilized to sort each of the various ILC subsets and examine their response to ILC subset-specific activators. Once established that the composition of human whole blood was largely enriched for ILC1s (with smaller numbers of ILC2s and ILC3s) and that these subsets (for ILC1s and ILC2s) could be activated to produce their “signature” cytokines, we were able to demonstrate cytokine production by ILC1s and ILC2s were differentially regulated by TGF-β and IL-10 and, in so doing, were able to reveal a novel cell intrinsic modulation of human ILC subsets.

## Materials and Methods

### Healthy adult volunteers

Whole blood samples were obtained from 89 healthy adult volunteers from the NIH’s Department of Transfusion Medicine, Clinical Center. De-identified donor information was available for 58/89 adult volunteers and is provided in Table 1. Data analysis was performed on whole blood samples that contained >1 ILC per 10,000 total cells acquired based on a desired CV of 5%, given the frequency of ILCs was ~0.09% of CD45+ cells in whole blood.

**Table 1:** Demographic of Healthy Adult Volunteers for Whole Blood Analysis of ILC subsets.

	All Samples			Sample with (>10 Total ILCs)		
	Total	Female	Male	Total	Female	Male
Sample size	58	19	39	48	15	33
Median age, (range)	49.5 (20–76)	50 (22–76)	48 (20–75)	51 (20–76)	55 (22–76)	49 (20–65)
Race						
Caucasian	37	12	25	31	9	22
African American	16	5	11	13	4	9
Asian	3	1	2	2	1	1
Other Hispanic	00	00	00	10	00	00
Unknown Native American	10	00	10	10	00	10
Mixed Race	0	0	0	0	0	0
Other	0	0	0	1	0	0
Unknown	1	0	1	1	0	1

### Ethics statement

Blood products (whole blood, buffy coats and elutriated lymphocytes) used in this study were collected from healthy adult volunteer donors under a protocol approved by the Institutional Review Board (IRB) of the Department of Transfusion Medicine, Clinical Center, National Institutes of Health (NIH; IRB 99-CC-0168). All volunteers gave informed written consent. Furthermore, all methods included in this study were performed in accordance with the relevant guidelines and regulations.

### Flow cytometry

Whole blood (2–5 mL) diluted in RPMI 1640 (ThermoFisher) supplemented with 2% L-glutamine and 0.1uM of Brefeldin A/monensin (MilliporeSigma) was incubated for 4hrs at 37 °C. For the whole blood phenotyping, the diluted whole blood samples were stained with antibodies to CD45 (HI30), CD117 (104D2), CD127 (eBioRDR5), CRTH2 (BM16) and a Lineage cocktail (LIN) for 30 mins at room temperature (see Table 2) with the total ILCs being defined as LIN− CD45+ CD127+, the ILC1s as CD117− CRTH2−, ILC2s as CRTH2+ CD117−/+ and the ILC3s as CRTH2− CD117+. ILC3 subpopulations were evaluated in whole blood using the NKp44 (44.189) antibody to defined ILC3as as CRTH2− CD117+ NKp44− and ILC3bs as CRTH2− CD117+ NKp44+. Additionally, whole blood samples were stained to ascertain the surface expression of IL-10Rα (3F9; BioLegend) and IL-20Rα (173714; R&D Systems) on ILCs and CD14+ (61D3) monocytes. Data were acquired using a LSRFortessa™ flow cytometer (BD Biosciences) and analyzed by using FlowJo 10.4.0 software (Tree Star), where individual gates were established using fluorescence minus one (FMO) controls.

**Table 2:** ILC Staining Panel for Flow Cytometry.

	Target	Color	Clones	Source
Lineage Markers	CD3	eFluor®450	OKT3	eBioscience
	CD4	eFluor®450	RPA-T4	eBioscience
	CD8	eFluor®450	OKT8	eBioscience
	CD14	eFluor®450	61D3	eBioscience
	CD16	BV421	3G8	BioLegend
	CD19	eFluor®450	HIB19	eBioscience
	CD56	BV421	HCD56	BioLegend
	CD11c	eFluor®450	3.9	eBioscience
	CD11b	BV421	ICRF44	BioLegend
	FceR1a	eFluor®450	AER-37	eBioscience
	CD336 (NKp44)	PerCP-eFluor®710	44.189	eBioscience
ILC subset markers	CD45	Pac. Orange	HI30	Invitrogen
	CD117 (cKit)	BV650	104D2	BioLegend
	CD127	APC eFluor®780	eBioRDR5	eBioscience
	CD294 (CRTH2)	Alexa Fluor®647	BM16	Biolegend

### ***Ex vivo*** Innate Lymphoid Cell Stimulation

*Ex vivo* experiments were performed using cells isolated from buffy coats or elutriated lymphocytes of men and women age 20–57; with a median age of 41. ILCs were isolated by first isolating PMBCs using lymphocyte separation medium (LSM; MP Biomedicals) and then performing a red blood cell lysis using ACK lysing buffer (ThermoFisher), as needed, using a standard protocol^25^. The PBMCs were stained and sorted using flow cytometry for the following populations: total ILCs, ILC1s, ILC2s, and ILC3s, as phenotypically described above, using a FACSAria™ II cell sorter (BD Biosciences). ILCs were sorted for yield with a typical purity of >95% for ILC1, >95% for ILC2 and >85% for ILC3 populations. Post sorting viability was consistently >95%. Total or individual ILC subsets (2 × 10^3^) were then cultured in 96-well round bottom plates in X-Vivo™ 15 medium (Lonza) supplemented with 1% heat-inactivated human AB serum, 10 U/mL IL-2 or Proleukin® (PeproTech or Prometheus Laboratories) and 50 ng/mL rIL-7 (PeproTech). ILC subsets were stimulated with ILC activating cytokines: 50 ng/mL of IL-12 (R&Dsystems) and IL-15 (PeproTech) for ILC1 activation, 50 ng/mL of IL-25 (PeproTech) and IL-33 (PeproTech) for ILC2 activation, 50 ng/mL of IL-1β and IL-23 (R&Dsystems) for ILC3 activation, or with 125/1,250 pg/uL of PMA/ionomycin (MilliporeSigma) at 37 °C. For defining any immunoregulatory roles, ILCs were stimulated in the presence or absence of 50 ng/mL of IL-10 or TGF-β (PeproTech).

### Cytokine measurements

Culture supernatants were collected at days 2, 4, and/or 5 and assessed using the MILLIPLEX® _MAP_ Human Th17 Magnetic Bead Panel (Millipore Sigma) customized for 10-analytes: IL-4, −5, −6, −9, −10, −13, −17A, −22, IFN-γ and TNF-α). The assay was performed according to the manufacturer’s instructions. Sample detection was performed using a Luminex® instrument, Bio-Plex® MAGPIX® Multiplex Reader (Bio-RAD) followed by data acquisition and management using the following software: xPONENT® 4.2 system, Bio-Plex Manager™ MP and Bio-Plex Manager™ 6.1.

### RNA isolation and qPCR

Sorted ILCs (n = 9) and PMBCs (n = 7) isolated from healthy adult volunteers were stored in RLT buffer at −80 °C prior to RNA preparation, using the RNeasy Mini Kit (Qiagen). Isolated RNA (<2ug) was used to generate cDNA using the qScript cDNA supermix (Quantabio). The resulting cDNA was then complexed with the TaqMan® assay targets for IL-10Rα, TGF-βRI, IL-20Rα and endogenous controls 18S or GAPDH using TaqMan® Fast Advanced Master Mix based on the manufacturer’s instructions. The ViiA™ 7 system (Applied Biosystems) was used to perform the thermocycling. Threshold cycle (CT) value for each gene and the endogenous control was used to determine the relative transcript levels (1/ΔCT), where ΔCT is the difference between the CT of the target gene and the corresponding endogenous control for each donor.

### Statistical analysis

All statistical analysis was performed using GraphPad Prism (v. 7.0c). Unless otherwise stated, the geometric mean (GM) was used as a measure of central tendency. The Mann Whitney test was used to compare groups and the Wilcoxin test was used in the analysis of paired samples.

## Results

### Frequency and distribution of ILC subsets in the peripheral blood of healthy volunteers

**Figure 1:**
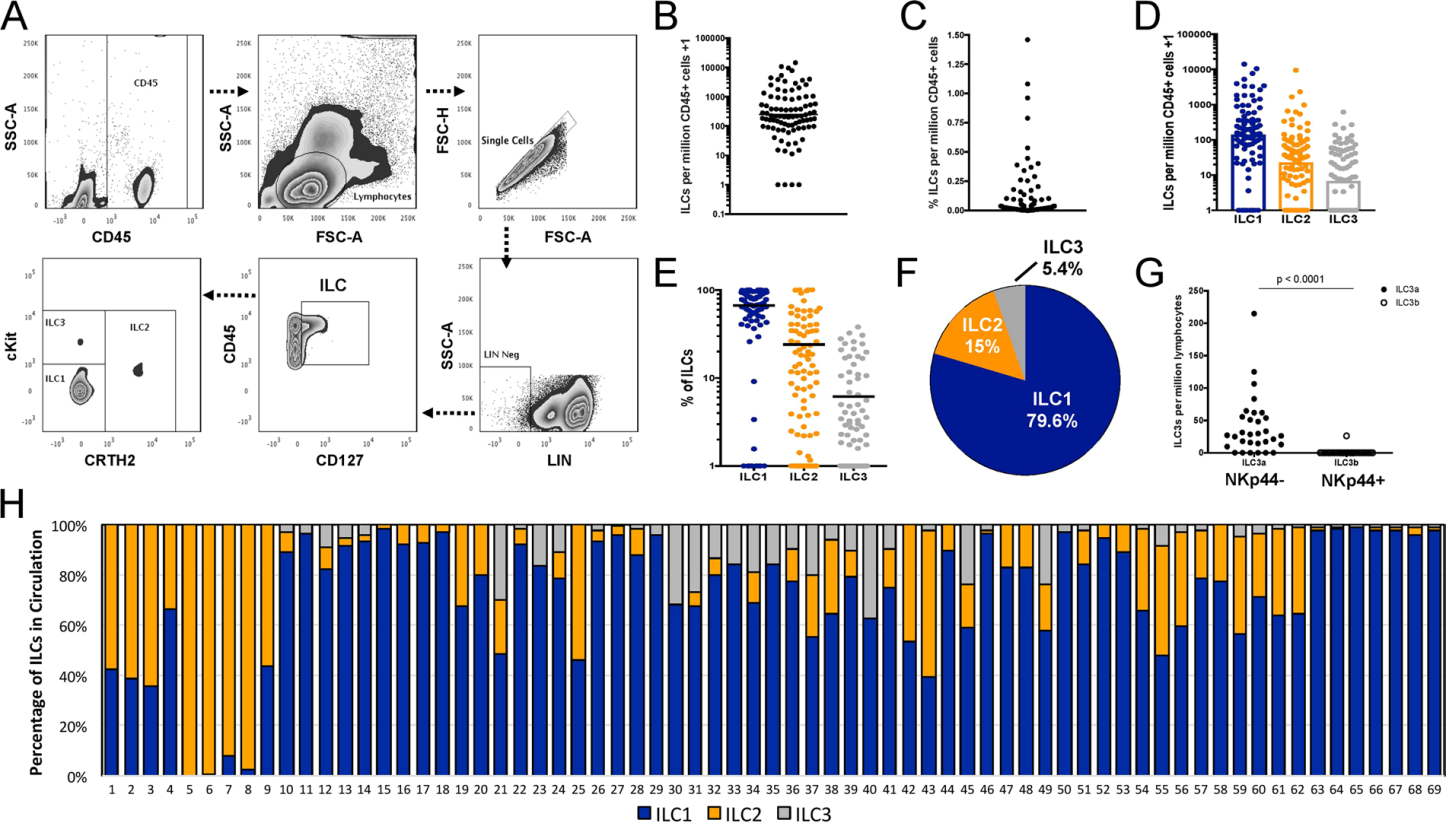
Frequency and distribution of ILC subsets in the peripheral blood of healthy volunteers. (**A**) Gating strategy for total ILCs ((CD45+ Lin-CD127+), ILC1s (CD45+ Lin-CD127+ cKit-NKp44-), ILC2s (CD45+ Lin-CD127+ CRTH2+) and ILC3s (CD45+ Lin-CD127+ cKit+ NKp44−/+). Panels are representative of multiple independent experiments (n = 89). (**B**) The frequency of total ILCs per million CD45+ cells and (**C**) the percentage of total ILCs per million CD45+ cells. Each dot represents an individual donor (n = 89) and the horizontal lines are the GM. ILC surface markers were used to quantify (**D**) the frequency of ILC1 (blue dots), ILC2 (orange dots) and ILC3 (gray dots) per million CD45+ cells and (**E**) the percentage of ILC subsets per total ILC cells in 69 donors. (**F**) ILC3a (CD45+ Lin-CD127+ cKit + NKp44-) and ILC3b (CD45+ Lin-CD127+ cKit+ NKp44+) frequencies per million lymphocytes in the peripheral blood. (**G**) Stacked bars individual ILC subsets by individual donor (n = 69).

To determine the frequency and distribution of ILC subsets in healthy human subjects, whole blood was taken from healthy adult volunteers (n = 89) and the ILC subsets present in peripheral blood were quantified based on surface marker staining using the strategy shown in Fig. 1A. By first gating on the total ILC population (defined as single cells that were CD45+ Lin-CD127+), these cells could then be divided into their respective ILC subsets as follows: ILC1s (CD45+ Lin− CD127+ cKit-NKp44−), ILC2s (CD45+ Lin-CD127+ CRTH2+) and ILC3s (CD45+ Lin− CD127+ cKit+ NKp44−/+). ILC quantification by donor revealed that 96% (85/89 donors had detectable ILC numbers. The GM number of total ILCs per million CD45+ cells found in the circulation was 246.7 (range 1 to 14,599; Fig. 1B) a number that translated to a geometric mean (GM) frequency of 0.09% (range 0 to 1.46%), of total CD45+ cells (Fig. 1C). The influence of donor variables such as sex, race and age were also examined in a subgroup of donors (n = 58) for whom data were available. The frequency of total or ILC subsets per million CD45+ cells in the peripheral blood were largely unaffected by the sex, race, or age of the donor (Figure S1). However, donor age did negatively correlate with the frequency of ILC2s found in circulation (Figure S1C). Therefore, ILCs were largely detectable in the circulation of healthy adult volunteers albeit at low frequencies.

Given the rarity of ILCs in the circulation, a minimum of 10 total ILCs per 10,000 acquired was used during analysis to establish greater confidence. In total, 69/89 (77.5%) of the donors had 10 or greater total ILCs per 10,000 acquired cells; the remaining donors were excluded from ILC subset analyses. Among the 69 donors evaluable, the GM of ILC subsets per million CD45+ cells in circulation was determined to be 141 for ILC1s, 23 for ILC2s and 6 for ILC3s (Fig. 1D). Overall, the GM frequencies of ILCs in the blood was 79.6% for ILC1s, 15.0% for ILC2s, and 5.4% for ILC3s (Fig. 1E). ILC3 subsets were also analyzed in a subgroup of donors (n = 31). The expression of NKp44 was used to delineate ILC3a (NKp44-) and ILC3b (NKp44+) subsets. The ILC3s in the circulation were found to be predominately ILC3as and not ILC3bs (Fig. 1F). Overall, the distribution of ILC in healthy adult volunteers was quite variable (Fig. 1G); however, these data indicate that while all ILC groups were detected in the blood, ILC1s were the most abundant ILC group in circulation.

### ILC1 and ILC2s produce large quantities of their signature cytokines when activated ***ex vivo***, while ILC3s produce non-signature cytokines when activated

Total ILCs and ILC subsets were isolated from the blood of healthy adult donors and stimulated *ex vivo* to determine the cytokine potential of the various cell types under different conditions. Total ILCs were cultured in media alone or under ILC activating conditions; IL-12/IL-15 for ILC1s, IL-25/IL-33 for ILC2s, IL-1β/IL-23 for ILC3s or following the addition of PMA/ionomycin; supernatants were subsequently assessed for production of cytokines: IL-4, IL-5, IL-9, IL-10, IL-13, IL-17A, IL-22, IFN-γ and TNF-α. With the exception of IL-13, cytokine production by total ILCs cultured in media alone (with IL-2 and IL-7) released little to no cytokines after culturing the cells for 2 and 4 days (Figure S2). Following stimulation with activating cytokines, cytokine levels appeared somewhat higher at 4 days than after 2 days (Table 3). However, stimulation with the various ILC activating conditions did not yield striking increases in the ILC signature cytokine levels. Total ILCs stimulated with PMA/ionomycin did yield more than 2-fold increases in IFN-γ, TNF-α and IL-17A (Table 3). Overall, these findings demonstrate that total ILCs when stimulated under various ILC activating conditions are unable to produce the robust and subset specific cytokine response commonly associated with activated ILCs.

**Table 3:** Cytokine Profile of *Ex vivo* Stimulated Total ILCs.

	Stimulation	Geometric Mean of Cytokine Fold Change (Range)*							
		IL-4	IL-5	IL-9	IL-10	IL-13	IL-17A	IFN-γ	TNF-α
Day 2	IL-12/IL-15	1.00 (1.00–1.08)	0.89 (0.80–0.99)	0.97 (0.86–1.12)	1.14 (10.92–1.67)	0.96 (0.94–0.99)	0.88 (0.60–1.00)	1.35 (1.00–2.51)	1.02 (0.00–1.06)
	IL-25/IL-33	1.02 (1.01–1.03)	1.77 (1.24–2.37)	1.79 (1.35–2.43)	1.46 (1.27–1.71)	1.16 (1.06–1.29)	1.37 (0.60–1.93)	1.05 (0.61–2.02)	1.73 (1.49–2.12)
	IL-1β/IL-23	1.00 (1.00–1.00)	1.56 (1.21–1.88)	1.40 (1.15–1.74)	1.13 (0.93–1.47)	1.12 (1.08–1.20)	1.14 (0.60–2.78)	2.08 (1.68–2.57)	1.94 (1.54–2.44)
	PMA/Ionomycin	1.17 (1.09–1.31)	0.84 (0.55–1.22)	1.96 (1.14–3.03)	1.09 (0.80–1.45)	0.98 (0.86–1.12)	2.54 (1.32–3.44)	2.79 (1.83–3.66)	2.87 (2.18–3.63)
Day 4	IL-12/IL-15	0.99 (0.97–1.00)	0.69 (0.30–1.16)	0.92 (0.44–1.50)	1.57 (1.00–2.65)	0.97 (0.87–1.10)	1.00 (1.00–1.00)	2.19 (1.00–3.99)	1.21 (0.39–2.17)
	IL-25/IL-33	1.04 (1.00–1.11)	2.43 (1.49–4.82)	1.74 (0.76–2.96)	2.89 (1.66–4.10)	1.32 (1.20–1.60)	1.17 (1.00–1.55)	1.64 (1.00–3.70)	1.66 (0.65–2.39)
	IL-1β/IL-23	1.00 (0.98–1.04)	2.28 (1.34–4.75)	1.59 (0.63–3.33)	1.99 (1.11–3.43)	1.25 (1.07–1.63)	1.39 (1.00–2.32)	3.33 (2.60–4.21)	2.15 (0.78–3.22)
	PMA/Ionomycin	1.17 (1.00–1.47)	1.05 (0.35–3.15)	2.28 (1.09–3.37)	1.21 (0.68–2.06)	1.09 (0.82–1.29)	2.34 (1.00–3.25)	4.12 (2.65–5.14)	3.36 (1.62–4.37)

*Fold changes were calculated using the ILC cultured in media alone condition, for each day respectively.

**Figure 2:**
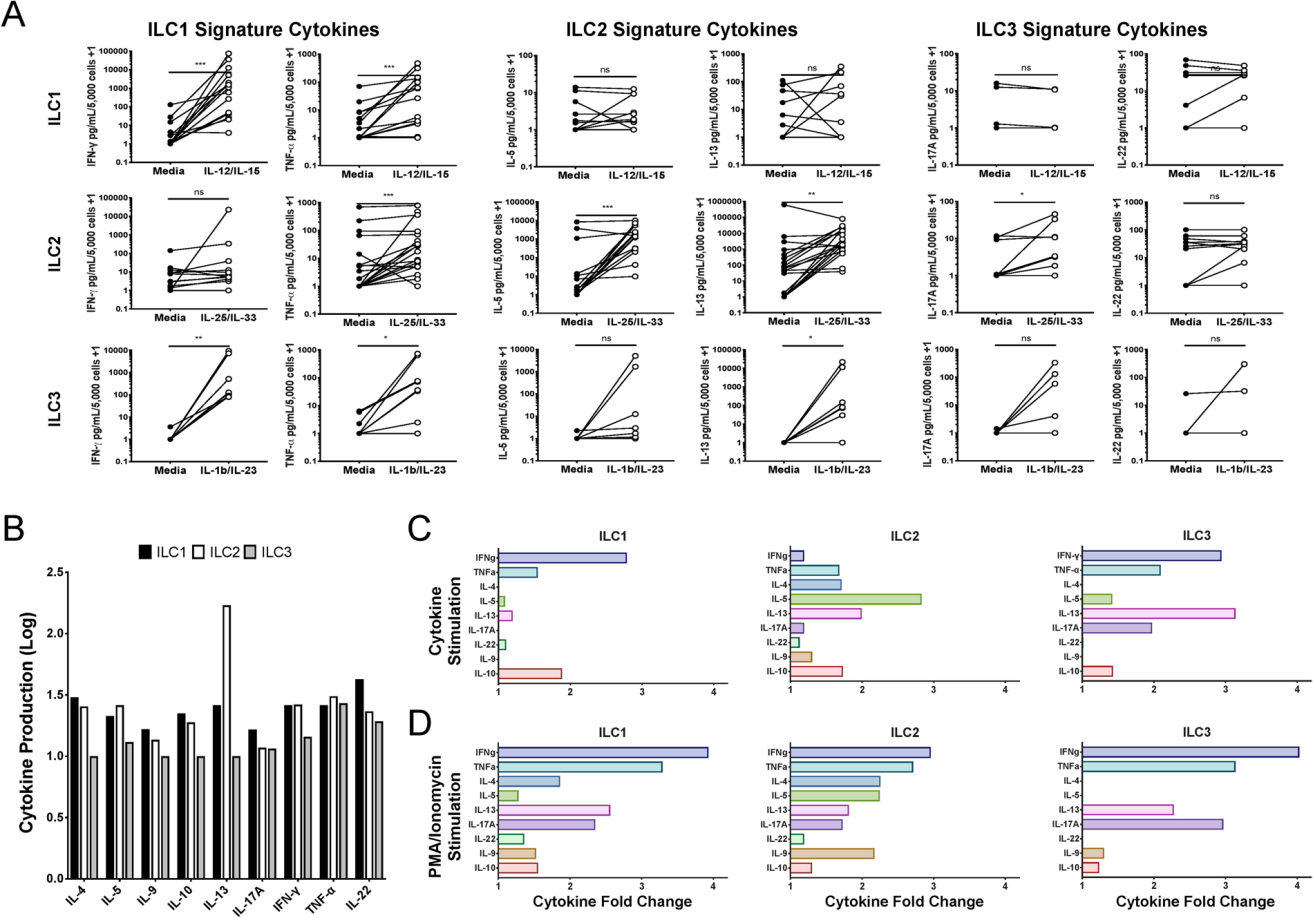
ILC1 and ILC2s produce large quantities of their signature cytokines when activated *ex vivo*, while ILC3s produce non-signature cytokines when activated. Panel A. The levels of IFN-γ, TNF-α, IL-5, IL-13, IL-17A, and IL-22 produced following stimulation (open circles) under ILC1 conditions (IL-12/IL-15) (top row), ILC2 conditions (IL-15/IL-33) (middle row), or ILC3 conditions (bottom row) or cultured in absence of stimulation (media – closed circles. Data are from multiple experiments with n = 15 for ILC1, n = 19 for ILC2 and n = 8 for ILC3 groups. Levels of significance are indicated by ***p < 0.001, **p < 0.01, *p < 0.05, or ns, not significant, as ascertained using the Wilcoxon pair-wise comparison. Panel B. Sorted ILC subsets from a subset of donors from A (n = 10, 14, and 4 for ILC1s, ILC2s, and ILC3s respectively) were individually cultured in media alone, ILC activating cytokines, or PMA/ionomycin for 5 days. Production of IL-4, IL-5, IL-9, IL-10, IL-13, IL-17A, IL-22, IFN-γ and TNF-α was assessed. (Panel B) Geometric mean cytokine production in pg/ml for unstimulated sorted ILC1 (black bars), ILC2 (white bars), and ILC3 (gray bars). The GM fold change in cytokine production over unstimulated cells for sorted ILC1 (left panel) sorted ILC2 (middle panel) and ILC3 (right panel) after being activated with ILC-subset specific ILC activating cytokines (Panel C) and following PMA/ionomycin stimulation (Panel D).

In contrast to total ILCs, *ex vivo* analysis of sorted ILC subsets revealed high levels of ILC signature cytokines when stimulated with the various ILC activating conditions. *Ex vivo* stimulation of ILC1s with IL-12/IL-15 resulted in a GM 223-fold increase in IFN-γ production and a GM 6-fold increase in TNF-α production when compared to ILC1s cultured without stimulation (Fig. 2A). ILC2s stimulated with IL-25/IL-33 produced a GM 200-fold increase in IL-5 and a GM 46-fold increase in IL-13 when compared to ILC2s cultured without stimulation. Moreover, stimulation of ILC2s also resulted in a 4-fold increase in TNF-α. Stimulation of ILC3s with IL-1β/IL-23 did not result in a statistically significant production of ILC3 signature cytokines IL-17A or IL-22, although a 7- and 2-fold increase was observed for both cytokines respectively when compared to ILC3s cultured without stimulation. Interestingly, stimulation of ILC3s resulted in a significant increase in ILC1 signature cytokines IFN-γ (311-fold increase), TNF-α (23-fold increase) and the ILC2 signature cytokine IL-13 (94-fold increase) when compared to ILC3s cultured without stimulation (Fig. 2A,C). Taken together, these findings demonstrate that *ex vivo* stimulation of ILC1s and ILC2s largely results in production of their signature cytokines while ILC3s produce only modest amounts of their signature cytokines but significantly larger amounts of IFN-γ, TNF-α and IL-13.

Additional analysis of the cytokine profile of ILC subsets cultured without stimulation revealed ILC subsets in culture produced little to no measurable cytokines. ILC2 production of IL-13 (GM = 2,230 pg/mL) was the only cytokine detected when ILCs were cultured in the absence of stimulation for 5 days (Fig. 2B). Stimulation of ILC subsets with their respective activating cytokines resulted in an increase in largely signature cytokines, with the exception of ILC3s that produced predominately non-signature cytokines (Fig. 2C). Analysis of individual donors revealed little variation between donors for ILC subsets stimulated with cytokines (Figure S3A) or PMA/ionomycin (Figure S3B). Moreover, stimulation of ILC subsets with PMA/ionomycin resulted in a broad cytokine activation profile with particular enrichment for ILC signature cytokines (Fig. 2D).

### ILC1 and ILC2s are a source of the immunoregulatory cytokine IL-10 when activated ***ex vivo***

**Figure 3:**
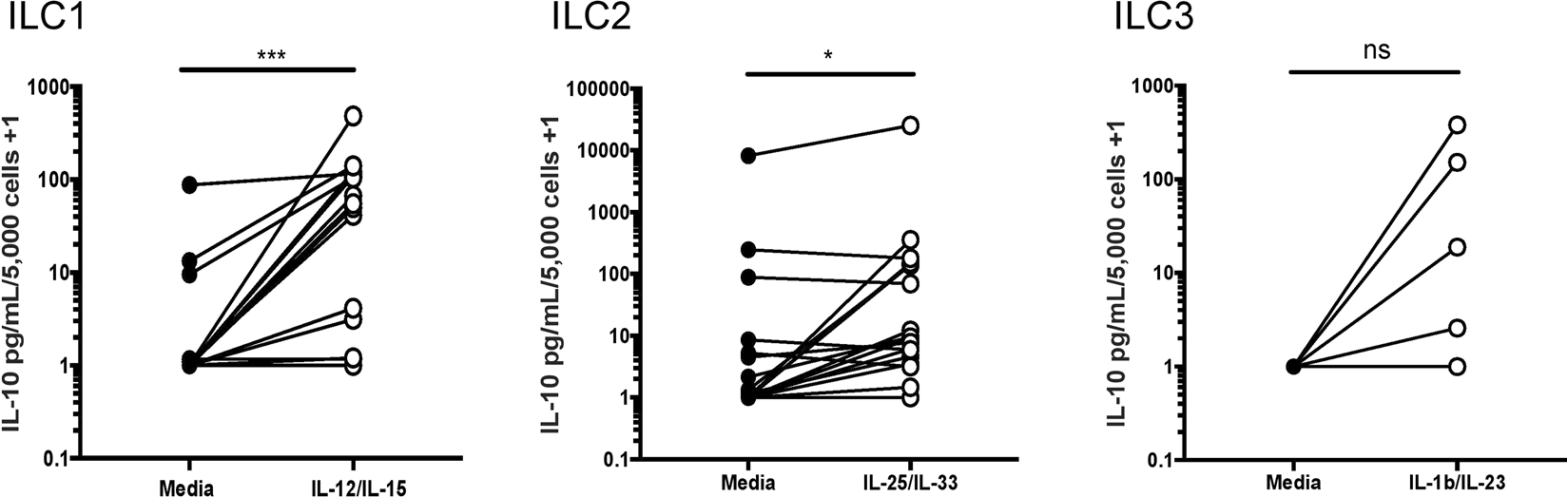
ILC1 and ILC2s are a source of the immunoregulatory cytokine IL-10 when activated *ex vivo*. Sorted ILC subsets (2,000 ILCs/well) were stimulated in the presence of 50 ng/mL of ILC activating cytokines (IL-12/IL-15 for ILC1s, IL/25/IL-33 for ILC2s and IL-1β/IL-23 for ILC3 subsets) for 5 days. The culture supernatants were then harvested from ILC subsets in media alone or following stimulation and then evaluated for the immunoregulatory cytokine IL-10 as part of a multiplex Luminex analysis. Data are representative of multiple experiments with n = 15 for ILC1, n = 19 for ILC2 and n = 8 for ILC3 groups. Paired comparisons were accomplished using the Wilcoxon test and levels of significance are indicated as: ***p < 0.001, *p < 0.05, ns, not significant.

Analysis of ILC subsets following cytokine activation revealed a marked-increase in production of the immunoregulatory cytokine IL-10 from supernatants of stimulated ILC1s and ILC2s (Fig. 3). Stimulation resulted in a 13-fold increase in IL-10 production by ILC1s (p < 0.001) and an approximately 6-fold increase by ILC2s (p < 0.05) when compared to ILC subsets cultured without stimulation. ILC3s showed a similar but not significant 6-fold increase. Furthermore, stimulation of total ILCs revealed a similar increase in IL-10 production when compared to unstimulated cells. Total ILCs stimulated with IL-25/IL-33 or IL-1β/IL-23 resulted in a 2-fold increase or greater in IL-10 cytokine levels after 4 days of culture (Table 3). These findings indicate that all ILC subsets are a potential source of IL-10 production following stimulation.

### IL-10 differentially modulates the cytokine production of activated ILC2s despite expression of IL-10R by all ILC subsets

**Figure 4:**
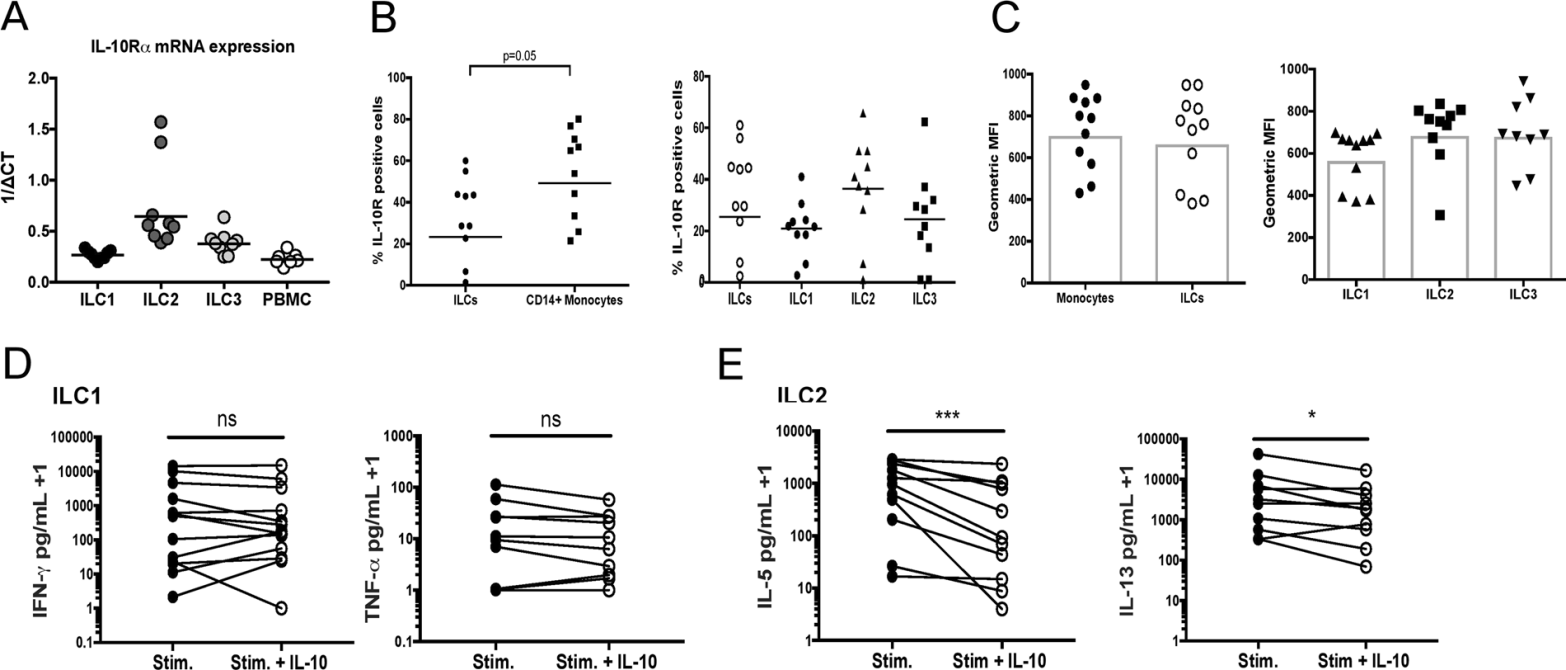
IL-10 differentially modulates the cytokine production of activated ILC2s despite expression of IL-10R by all ILC subsets. (**A**) Sorted ILC subsets (n = 9) and PBMCs (n = 7) were isolated from healthy volunteers and assessed for expression of IL-10R mRNA by qPCR. Data are shown as a combination of three independent experiments. (**B,C**) IL-10R expression on circulating monocytes and ILC subsets was assessed in the whole blood of healthy volunteers (n = 10) using by flow cytometry for (**B**) surface expression and (**C**) per cell intensity assessed by geometric mean fluorescence intensity (geometric MFI). (**D,E**) ILC1 (n = 13) and ILC2 (n = 10–11) subsets were sorted from healthy donors and stimulated with activating cytokines (IL-12/IL-15 for ILC1s and IL/25/IL-33 for ILC2s) in the presence or absence of 50 ng/mL of IL-10 for 5 days at 2,000 cells/well. The culture supernatants were assessed for production of (**D**) ILC1 and (**E**) ILC2 signature cytokines. Paired comparison of stimulated and stimulated + IL-10 conditions was accomplished with the Wilcoxon test and levels of significance were indicated by: ***p < 0.0001, *p < 0.05 and ns, not significant.

To determine the ability of ILCs to be modulated by exogenous IL-10, ILCs were first assessed for IL-10R expression using qPCR. Sorted ILC subsets and PBMCs were indeed positive for IL-10Rα mRNA (Fig. 4A). All ILC subsets also expressed IL-10Rα protein (Fig. 4B) as detected by flow cytometry. However, the GM percent of cells positive for the surface expression of IL-10Rα in total ILCs was approximately 20%, significantly less than the percent positive for the CD14+ monocytes used as a control (Fig. 4B). ILC2s had the greatest percent of cells positive for the surface expression of IL-10Rα when compared to other ILC subsets while the percent positive for ILC1s and ILC3s was comparable. Despite the differences in the frequency of IL-10Rα positivity, monocytes and ILCs had a similar GM fluorescence intensity (MFI) of 654 and 629 respectively for IL-10Rα expression (Fig. 4C).

ILC1 and ILC2 subsets were then stimulated using their respective activating cytokines in the presence or absence of IL-10 to determine the effect of the immunoregulatory cytokine on ILC activation. ILC1 production of the ILC1 signature cytokines IFN-γ and TNF-α was not significantly altered by the addition of IL-10 (Fig. 4D). However, ILC2 production of the ILC2 signature cytokines IL-5 (p < 0.0001) and IL-13 (p < 0.05) was significantly inhibited, following activation in the presence of IL-10 (Fig. 4E). Taken together, these data demonstrate that although all ILC subsets express IL-10Rα, the inhibitory effects of IL-10 selectively modulated ILC2 cytokine production and had no effect on ILC1 cytokine production.

Examination of additional immunoregulatory family members was extended to include the IL-20 family of cytokines, which are also members of the IL-10 superfamily. The expression of IL-20Rα, a receptor shared by multiple IL-20 family members, was examined in all ILC subsets by both qPCR and flow cytometry. IL-20Rα mRNA expression (Figure S4A) was undetectable in ILC subsets and a significantly lower number of ILCs in circulation were positive for IL-20Rα when compared to PBMCs (Figure S4B). The geometric MFI of IL-20Rα was also significantly lower on ILCs than PBMCs (Figure S4C).

### ILC1 and ILC2 activity is also differentially regulated by TGF-β

**Figure 5:**
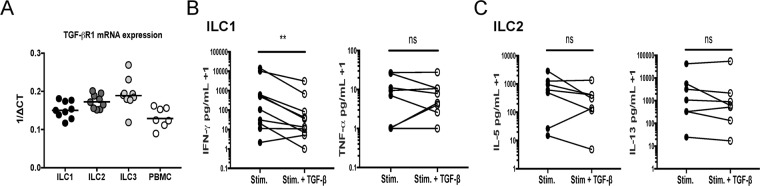
ILC1 and ILC2 activity is also differentially regulated by TGF-β. (**A**) Sorted ILC subsets (n = 9) and PBMCs (n = 7) were isolated from healthy volunteers and assessed for TGF-βRI expression by qPCR. Activated ILC1 (n = 10) and activated ILC2 (n = 7) subsets were stimulated in the presence or absence of 50 ng/mL of TGF-β for 5 days at 2,000 cells/well. The culture supernatants were then analyzed for ILC signature cytokine production (**B**) IFN-γ and TNF-α for ILC1s and (**C**) IL-5 and IL-13 for ILC2s. Paired comparison of stimulated and stimulated + TGF-β conditions was accomplished with the Wilcoxon test and levels of significance were indicated by: **p < 0.001 and ns, not significant.

In addition to IL-10, the regulatory effects of TGF-β on ILC1 and ILC2 activation were also evaluated. Quantitative real-time PCR was used to assess TGF-βR1 mRNA expression in ILC subsets and PBMCs from healthy donors. All ILC subsets expressed TGF-βR1 mRNA at levels similar to those expressed in PBMCs (Fig. 5A). *Ex vivo* stimulation of ILC1s in the presence of TGF-β resulted in the marked reduction of the ILC1 signature cytokine IFN-γ but had no effect on TNF-α (Fig. 5B). However, ILC2 stimulation with TGF-β had no effect on the production of ILC2 signature cytokines (Fig. 5C). These data demonstrate that, in marked contrast to the findings for IL-10, TGF-β modulated ILC1 cytokine production but not that of ILC2s following *ex vivo* activation (Fig. 5C).

## Discussion

Analysis of the frequency and distribution of circulating ILC populations in healthy North American adult volunteers verified that, in a larger cohort of patients, ILCs are found in extremely low frequencies accounting for, on average, 344 ILCs for every million leukocytes (as defined by CD45 positivity). The distribution of ILC subsets in the peripheral blood revealed an abundance of ILC1s and a smaller proportion of ILC2s and ILC3s. *Ex vivo* stimulation of sorted ILC subsets found in peripheral blood resulted in the prototypical response: the robust production of IFN-γ and TNF-α by activated ILC1s as well as IL-5 and IL-13 by activated ILC2s. Surprisingly, *ex vivo* stimulation of ILC3s resulted in the robust production of non-signature cytokines. This study also revealed that ILC subsets in circulation expressed receptors for IL-10 and TGF-β. *Ex vivo* stimulation of ILC1s in the presence of TGF-β resulted in an inhibition of IFN-γ, while IL-10 had no effect. In contrast, *ex vivo* production of IL-5 and IL-13 by ILC2s was significantly abrogated by the presence of IL-10 but not TGF-β. These findings demonstrate that ILCs found in the circulation of healthy volunteers when stimulated with their activating cytokines *ex vivo* produce large amounts of cytokines that can be differentially regulated by the immunoregulatory cytokines IL-10 and TGF-β.

Characterization of the frequency and composition of ILC subsets in the circulation of healthy individuals revealed donor variability in both the number of total ILCs and ILC subsets found in circulation but also the distribution of ILC subsets. Although deficiency in human ILCs has been described in patients with severe combined immunodeficiency (SCID)^26^, a small proportion of healthy individuals in our study cohort had no detectable ILCs. Interestingly, donor variables such as sex, age and race had largely no impact on the frequency of total ILCs or individual ILC subsets examined. Although, sex bias has been demonstrated for KLRG1- ILC2s in naïve mice of reproductive age, where a greater proportion of these cells was seen in female mice but virtually absent in male mice^27^, this seems not to be the case in humans. The distribution of ILC3 subpopulations in the circulation of healthy adults was validated by this study. Previous analysis of ILC3a and ILC3b populations in the peripheral blood revealed an absence of ILC3bs in healthy individuals; however, ILC3bs have been detected in the circulation of individuals with psoriasis^28^ and following allogeneic hematopoietic stem cell transplantation^29^. When examining ILC populations it is important to consider that both ILC subset quantification can be influenced by both ILC tissue specificity, resulting in varied surface marker expression^30^ and the host inflammatory state, resulting in changes in ILC migration or residency^31–33^.

*Ex vivo* stimulation of individual ILC subsets has provided a greater understanding of the functional capacity of human ILCs isolated from the peripheral blood. When sorted ILCs were cultured with IL-2 and IL-7 to promote survival and proliferation, cytokine production by either total ILCs or individual ILC subsets was minimal. The absence of spontaneous cytokine production suggests that ILC populations in peripheral blood are rather quiescent in nature. Stimulation of total ILCs under various ILC activating conditions or with a broad activator such as PMA/ionomycin demonstrated that ILCs isolated from the peripheral blood were capable of responding towards specific stimuli as well as in response to PMA/ionomycin activation. However, the magnitude of the signature cytokines released following activation of total ILCs with ILC activating cytokines was rather modest (Table 3). The ability of total ILCs to produce some but not all signature cytokines following appropriate stimuli indicates that cumulative cytokine production from total ILCs may be greatly influenced by the composition of ILC subsets or through the production of additional cytokines, particularly those with modulatory effects.

The examination of total ILC preparations highlights the importance of examining the cytokine capacity of individually sorted ILC populations. *Ex vivo* stimulation of ILC1s and ILC2s did result in the robust production of signature cytokines, as anticipated based on previous observations^34^. A multianalyte approach for cytokine detection was utilized to not only measure signature cytokines but to also identify additional cytokines secreted by ILCs following activation. Transcriptomic analysis of single-cell CD127+ ILCs, from tonsil tissue, revealed the heterogeneity present within each human ILC subset^35^. This study demonstrates that ILC1s and ILC2s activated with their respective activating cytokines exclusively produced large quantities of their signature cytokines. Stimulation of ILC1s and ILC2s with PMA/ionomycin revealed a broader profile of secreted cytokines demonstrating the capacity of individual ILC subsets to produce a wide range of cytokines. Furthermore, stimulation with PMA/ionomycin resulted in a greater magnitude of cytokine secretion than observed with cytokine activation alone (Fig. 2C,D).

The detection of IL-10 in culture supernatants following activation indicates that ILC1- and ILC2-derived IL-10 may further attenuate ILC activation. IL-10 production by ILCs has previously been described in ILC1s found in the lymph nodes, in peripheral tissue of naïve mice^36^ and in recent studies describing ILCreg in both murine and human tissues^2^. While ILC production of TGF-β was not assessed in this study, human ILCreg are poised to produce both IL-10 and TGF-β^2^. Taken together, distinct human ILC subsets may serve as a source of IL-10, that may work in a paracrine or autocrine manner (for ILC2s) to differentially modulate the production of ILC2 signature cytokines. In addition to ILCreg, activated ILC1s and ILC2s may also serve as a source of IL-10.

Surprisingly, the production of signature cytokines IL-17A and IL-22 by activated ILC3s was not observed. Given the phenotypic analysis of ILC3s in circulation revealed the predominance of ILC3as and not ILC3bs, the absence of IL-22 (a prototypical ILC3b cytokine) in culture supernatants was not surprising. This study demonstrates that although the cKit + CRTH2- ILC population found in circulation phenotypically resembles the ILC3 subset, specifically ILC3as, perhaps these cells have not yet acquired the lineage specificity exhibited in peripheral tissue to produce robust amounts of their signature cytokines, namely IL-17A or IL-22. Instead, IL-1β/IL-23 activated ILC3s produced significant amounts of IFN-γ, TNF-α, IL-13 and modest amounts of IL-17A when compared to unstimulated ILC3s. A similar cytokine profile was observed when ILC3s were stimulated with PMA/ionomycin. These results indicate that ILC3s found in circulation may be functionally naïve, requiring additional tissue specific signals, or represent a population of multi-potent human ILCs designated ILC precursors (ILCp) which have the potential to differentiate into distinct ILC subsets^37^. ILCp isolated from peripheral blood were incapable of producing ILC3 signature cytokines upon stimulation yet resulted in the expansion and differentiation of ILCp into multiple ILC subsets following prolonged exposure to IL-1β/IL-23^37^. Although, differences in staining and gating strategies make it difficult to make a direct comparison between the cKit + population described in this study and ILCp, many similarities do exist including their ability to produce non-signature cytokines and inability to produce robust amounts of ILC3 signature cytokines following activation.

To evaluate potential mechanisms by which the ILC activation is modulated, ILC subsets in circulation were examined for IL-10 and TGF-β receptor expression. Characterization of ILC subsets present during homeostasis revealed both gene and protein expression for IL-10Rα and gene expression for TGF-βR1. However, mRNA and protein level expression of IL-20Rα, a common signaling component for IL-10 superfamily members IL-19 and IL-24, was not readily detected in circulating ILC subsets under homeostatic conditions.

*Ex vivo* analysis of cytokine production following IL-10 and TGF-β exposure was only performed with the more abundant subsets (ILC1s and ILC2s) and not ILC3s, due to low frequency. ILC1 signature cytokine production was markedly reduced following simultaneous activation of ILC1s in the presence of TGF-β, but this effect was not observed in the presence of IL-10. Conversely, ILC2 activation and cytokine production was inhibited in the presence of IL-10 but not of TGF-β. Although significant differences in the number of positive cells or the intensity of IL-10Rα expression on ILC subsets was not observed, the ability of IL-10 to modulate cytokine production by ILC1s and ILC2s was drastically different. Recent findings have demonstrated that murine ILCreg selectively inhibit the activation of intestinal ILC1s and ILC3s, but not ILC2s, which did not express *Il10rb*^2^. The differential effects of ILCreg-derived IL-10 is indicative of the variability of IL-10 receptor expression on ILC subsets based on tissue specificity or following activation. Additional studies should be undertaken to determine if expression of various immunoregulatory receptors changes upon ILC activation.

Collectively, these data demonstrate that ILC1s and ILC2s found in the peripheral blood are largely poised to respond to appropriate stimuli, namely ILC activating cytokines, resulting in the secretion of signature cytokines as well as production of IL-10. Given the heterogeneity demonstrated for all human ILC subsets^30,35^, it is not surprising that stimulation with PMA/ionomycin revealed a broader cytokine profile for all ILC subsets than previously observed following cytokine stimulation. ILC3s isolated from peripheral blood exhibited a less differentiated state when activated resulting in the uncharacteristic production of ILC1- and ILC2-associated signature cytokines. Knowledge of the frequency and functional capacity of human ILC subsets in circulation is vital to understanding the function of these cells at homeostasis and in various inflammatory states. Furthermore, these findings provide an integral understanding of how ILCs are regulated and the extent to which IL-10 and TGF-β independently play a role. These findings may have important clinical implications for human diseases where uncontrolled cytokine production by ILCs may prove deleterious.

## Supplementary information

Dataset 1, Dataset 2, Dataset 3 and Dataset 4
