# Supplementary material for: Differential Modulation of Human Innate Lymphoid Cell (ILC) Subsets by IL-10 and TGF-β
Source: Sci Rep. 2019 Oct 4;9:14305. doi: 10.1038/s41598-019-50308-8 (PMC6778123; doi:10.1038/s41598-019-50308-8)
Supplement: Supplementary file 1 — Dataset 1, Dataset 2, Dataset 3 and Dataset 4 [file 41598_2019_50308_MOESM1_ESM.pdf]

Differential Modulation of Human Innate Lymphoid Cell (ILC) Subsets by IL-10 and TGF-β.

Sandra Bonne-Année*, Mabel C. Bush and Thomas B. Nutman

Helminth Immunology Section, Laboratory of Parasitic Diseases, National Institute of Allergy and Infectious Diseases, National Institutes of Health,

9000 Rockville Pike, Bethesda, MD 20892, USA

*Corresponding Author

Phone: 301-496-5399

Fax: 301-480-3757

e-mail: sandra.bonne-annee@nih.gov

This work was supported by Division of Intramural Research (DIR) of the National Institute of Allergy and Infectious Diseases, National Institutes of Health.

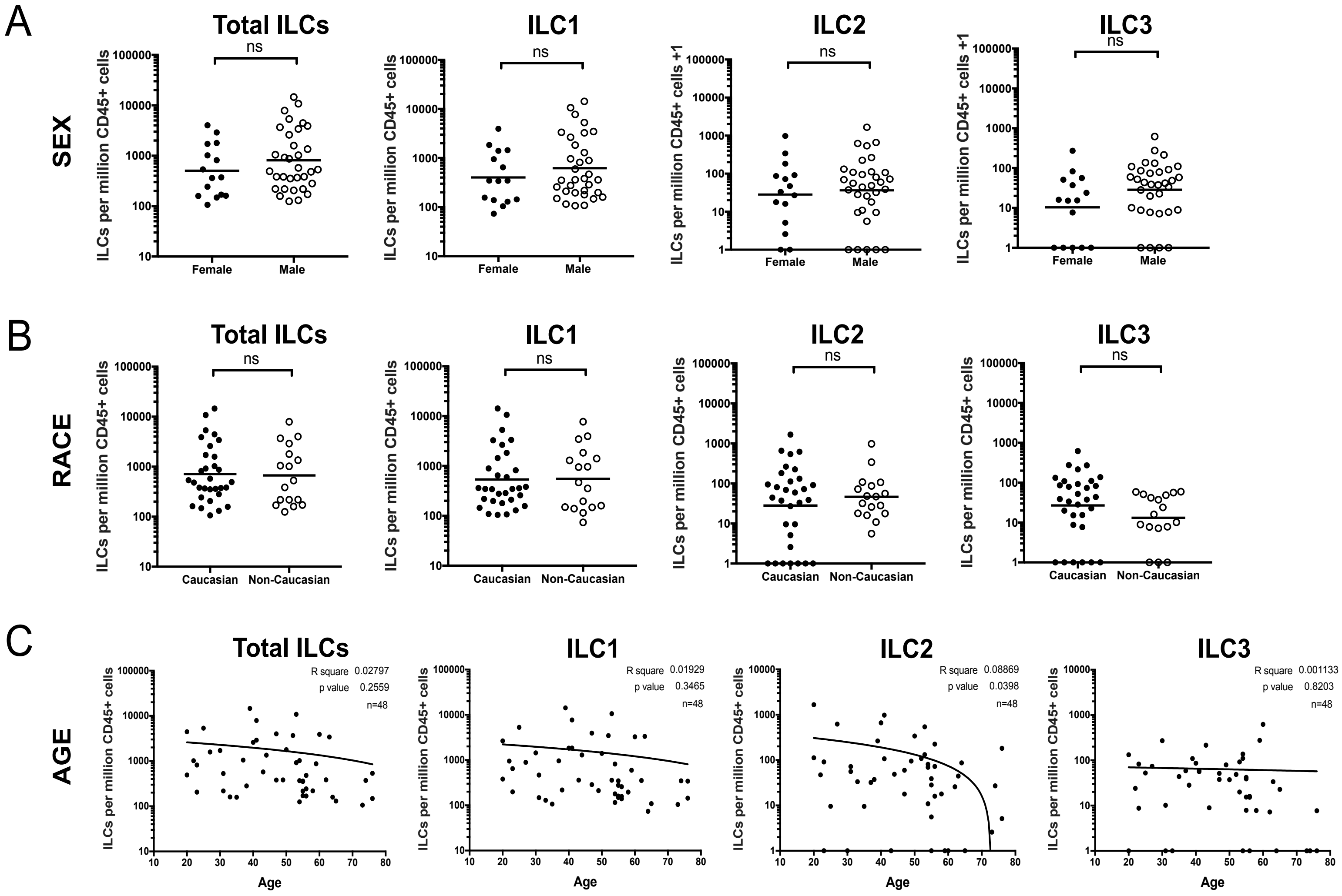

Figure S1. ILC frequencies in circulation are largely unaffected by donor sex, race or age. Whole blood samples from healthy volunteers (n=48) were stained for total ILCs (CD45+Lin-CD127+), ILC1s (CD45+Lin-CD127+cKit-NKp44-), ILC2s (CD45+Lin-CD127+CRTH2+) and ILC3s (CD45+Lin-CD127+cKit+NKp44-/+) and analyzed by flow cytometry. The frequency of total ILCs and individual ILC subsets per million (CD45+) leukocytes was examined in the context of the donor variables (**A**) sex (**B**) race and (**C**) age for which a linear regression model was performed. A paired comparison of (**A**) females and males and (**B**) Caucasian and Non-Caucasians was accomplished with the Wilcoxon test and levels of significance were indicated by: ns, not significant.

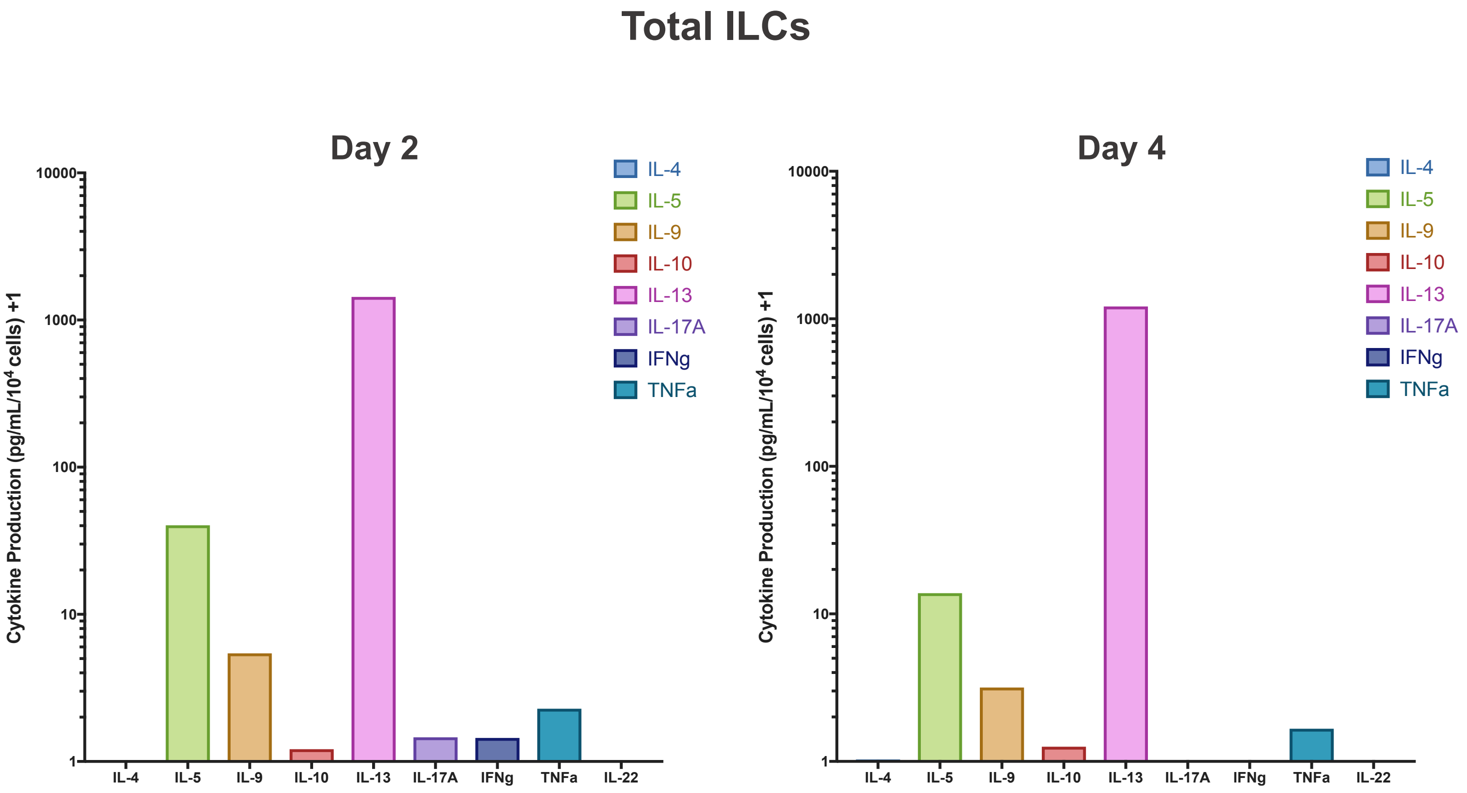

Figure S2. Cytokine production by total ILCs maintained in culture is minimal.

Total ILCs (CD45+Lin-CD127+) were sorted from peripheral blood products (n=4-7) using flow cytometry and maintained in culture media, supplemented with lL-2 and IL-7. Total ILC culture supernatants were then collected after 2 and 4 days of culture. The supernatants were then examined using a Luminex® multiplex assay (for IL-4, IL-5, IL-9, IL-10, IL-13, IL-17A, IL-22, IFN-γ and TNF-α) to determine the cytokine profile of total ILCs at baseline. Bars represent the geometric means of the data.

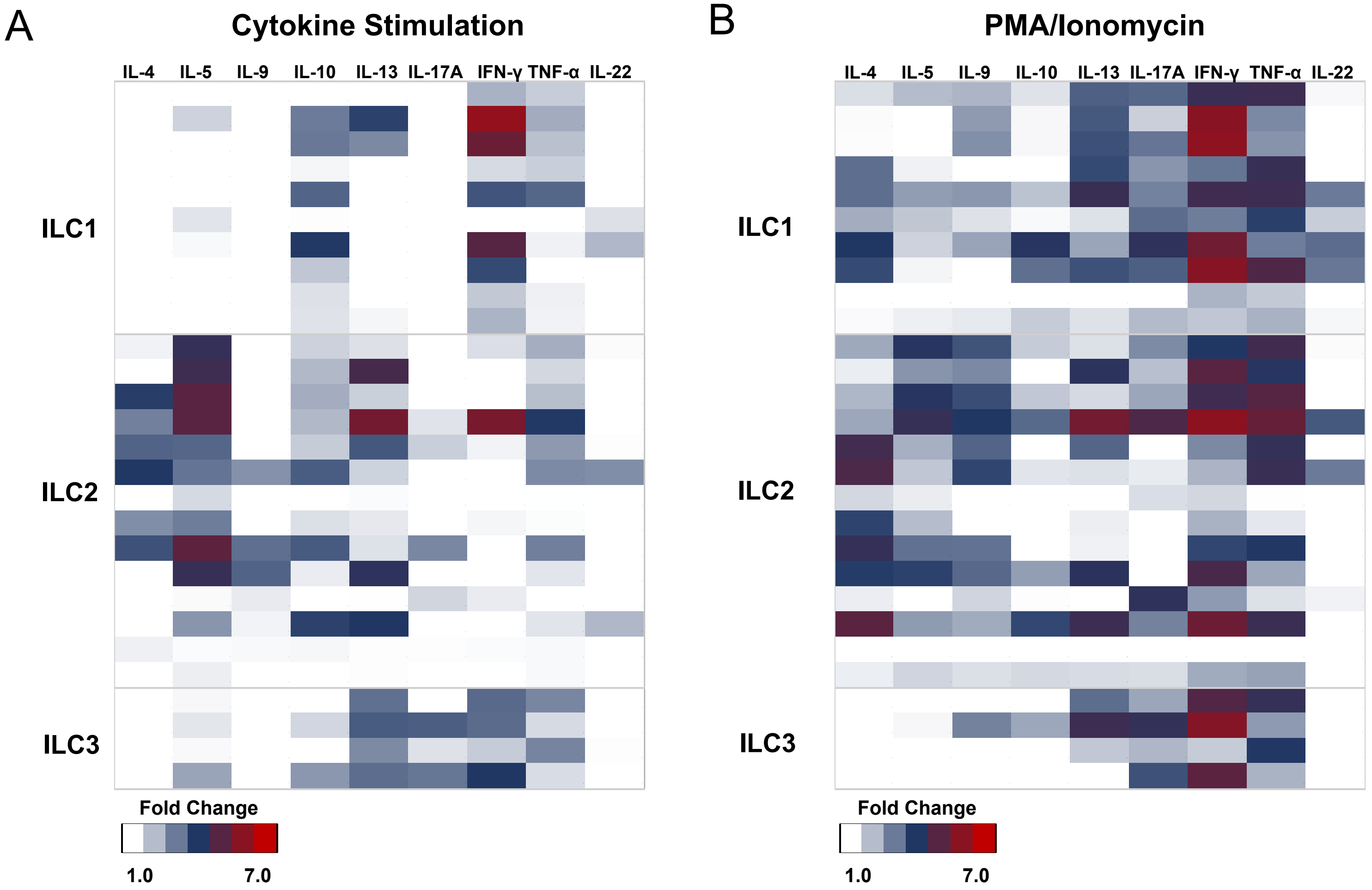

Figure S3. ILC activation by PMA/Ionomycin expands cytokine production by ILC subsets. Sorted ILC subsets (2,000 cells/well) were stimulated with cytokines for a total of 5 days in the presence of ILC activating cytokines (IL-12/IL-15 for ILC1s, IL-25/IL-33 for ILC2s and IL-1β/IL-23 for ILC3 subsets) or PMA/ionomycin. The fold change over media alone was calculated for the cytokines: IL-4, IL-5, IL-9, IL-10, IL-13, IL-17A, IL-22, IFN-γ and TNF-α. A comparison of cytokine expression by donor was performed for each ILC subset stimulated by (**A**) ILC activating cytokines or (**B**) PMA/ionomycin. Data represents the fold change of multiple donors stimulated with cytokines (n=15 for ILC1s, n=19 for ILC2s and n=8 for ILC3s) and PMA/ionomycin (n=10 for ILC1s, n=14 for ILC2s and n=4 for ILC3s), where white represents a fold change in cytokine production of 1 and red represents a fold change in cytokine production of 7, when compared to ILCs cultured in media alone (unstimulated).

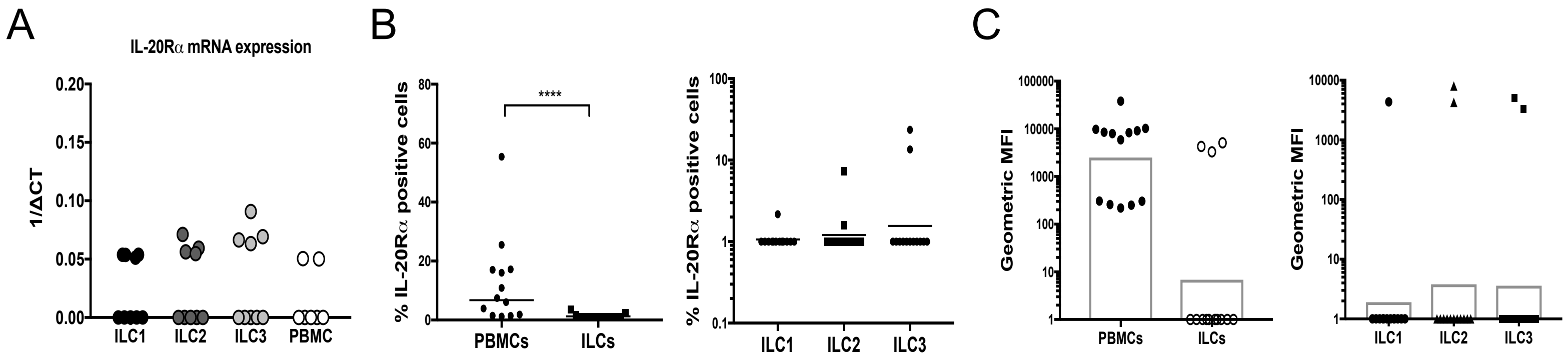

Figure S4. ILC subsets in circulation do not express IL-20Rα.

ILC expression of additional immunoregulatory receptors was examined by qPCR and flow cytometry for the IL-20Rα chain. (**A**) ILC subsets (n=9) and PBMCs (n=7) were isolated from healthy individuals and examined for gene expression IL-20Rα using qPCR. (**B-C**) ILCs and PBMC populations in whole blood samples (n=13) were stained and analyzed for (**B**) the percent of cells expressing IL-20Rα and (**C**) the geometric MFI of the receptor on each cell population. Data are representative of multiple experiments and the horizontal line and bars represent the geometric means. Groups were statistically compared using the Mann-Whitney test, ****, $p<0.00001$.
